# Exploring the value of lived experience in a mental health research centre

**DOI:** 10.1007/s00127-026-03167-4

**Published:** 2026-07-24

**Authors:** Rick Burgess, Alison Faulkner, Raza Griffiths, Rachel Hill, James Lane, Cassandra Lovelock, Bwalya Mulenga, Janahan Sivanathan, Rhea Sookdeosingh, Angela Sweeney, Sonia Thompson

**Affiliations:** ESRC Centre for Society and Mental Health, King’s College London, Alison Faulkner is based in London, London, UK

**Keywords:** Lived experience, Mental health research, Co-production, Organisational culture, Impact, Power, Barriers, Culture

## Abstract

This position paper has been collectively written and co-produced by the Lived Experience Advisory Board at the ESRC Centre for Society and Mental Health, along with a survivor researcher, lived experience researcher and lived experience facilitator. The paper highlights the value of lived experience involvement in mental health research. It asks what the barriers to ethical and meaningful involvement are and offers insights and advice about how they can be overcome. The paper was co-produced through facilitated reflective workshops that generated questions and themes based on the authors’ lived experience of mental health as well as their experiences of doing lived experience involvement work within universities, charities and other organisations. These reflections, along with scholarship on lived experience research, were used to write up the paper. The paper discusses six key aspects of doing lived experience involvement well: understanding what lived experience means, how to move from individual lived experience to impacting research, understanding the value lived experience brings to research, the nature of knowledge, the importance of power, and what research organisations need to hear and understand in order to meaningfully involve lived experience voices in their work. The paper ultimately concludes that the meaningful involvement of lived experience perspectives can have a profound impact on the quality and relevance of mental health research, but research organisations need to be committed and equipped to facilitate this involvement in ethical and equitable ways.

## Foreword

*It is a truth universally acknowledged*,* that an organisation with the stated goal of self-improvement*,* must be in want of a lived experience advisory board.* (adapted from Pride and Prejudice – Jane Austen)

Who amongst us can forget Mr Darcy’s original proposal? A handsome man of great means and status, who arrogantly threw a number of cutting insults in the direction of Elizabeth Bennett, even as he asked for her hand in marriage. He had already convinced himself that she would accept his offer and gave little thought to how she (a woman of less financial means and social standing) might receive such a proposition in the first place. As we are aware, Elizabeth did in fact refuse the impertinent offer and Mr Darcy went through a lot of angst, personal reflection and change before he proved worthy of betrothal to the protagonist of the story. And of course, they all lived happily ever after.

Back in the real world, organisations are courting people with lived experience (LE) in an endeavour to establish some sort of working relationship, that might be labelled coproduction or involvement or something like it. Whether as a result of funding requirements or new cultural expectations, we see more and more moves in the direction of this new type of relationship. We also unfortunately witness organisations acting as the 21st century personification of Mr Darcy when they put out their offer to form such a relationship.

But what if things were different. What if organisations had a commitment to real involvement from the start? What if they listened and leaned in to exploring lived experience and saw it as a boon rather than a hurdle? What if they resourced the people and the processes appropriately, so that they could benefit from that involvement? What if organisations were to prepare for involvement the same way that they prepare for other types of change processes? And what if we could all experience our own happily ever after, based on a more equal relationship, one which would be good for all of those concerned?

These are some of the issues raised in this position paper. It focuses on mental health lived experience and research but the issues raised can be extrapolated well beyond these confines. It certainly highlights the potential of lived experience to bring about some of the changes that many have admitted that they so sorely need.

*The genesis of this paper came about as a reflection on experiences of trying to perform my role as a lived experience adviser within an organisation. It wasn’t the first and would certainly not be the last*,* rather dismissive interaction I had with someone outside the lived experience group and it gave me pause for thought. I had a number of reflections which I put down on paper and later brought to the Lived Experience Advisory Board (LEAB) at the ESRC Centre for Society and Mental Health (CSMH) for discussion. (Sonia Thompson*,* LEAB member)*

This position paper is the culmination of many hours of the LEAB working and thinking together as a community of practice about those issues. We have grown and developed together as we crystallised our wisdom through a co-productive process.

 This is of course one of the benefits of being inside a knowledge generating organisation; we as members of the LEAB can also contribute to the generation of knowledge.

*My desire to be involved with this paper came from seeing too many research questions that were not relevant let alone the solutions offered. From participating with the hope that my LE expertise would produce meaningful results yet seeing too many outsiders (with no idea of the real problem) define and try to fix the problem without centring the reality. Just noting but mining my knowledge and not respectfully using it. Allowing me to attend and give some input but not set the agenda nor direct it. I already had the data so more was not needed. I had a good idea of the potential solutions too and was prepared not just to lead the way and say what needed doing but respectfully showing how to do it whilst understanding the limitations*.

*In summary I had so much negative pain and suffering that was eating away at my life. I longed for a chance to get it all out*,* to explain it and turn it into positive action. Few could handle it and sit with the discomfort of my shocking words. If the faces did not say enough*,* the lack of appropriate response and action did. I was left embarrassed*,* frustrated and disappointed. But opportunities were lost.*

*I found the LEAB and in the words of my colleague “came home” The CSMH affords us opportunities to engage with those with influence*,* to confront the ones who need to listen and stop blocking change*,* as well as consolidate efforts with allies to add pressure and pushback more effectively and on a larger scale. (Rachel Hill*,* LEAB member)*

We are building a collective theory of change where we encourage research to move beyond data collection to strategy and link our raw evidence to advocacy.

We work naturally and authentically and are allowed to disagree. We are not just expected to rubber stamp decisions. Our clashing, ugly and unique truths aren’t mistakes—they’re part of the story and the solution. When we work together, we tap into our strengths, share our ideas and values and act together for the people who matter most. We are a powerful and passionate group of experts with full human experience. We aim to be the light in the darkness that others can look up to and follow to achieve similar positive outcomes.

Between us we have over 70 years of experience of sitting in decision making groups where our lived experience of mental distress has been the reason for our recruitment. But we are also leaders, managers, volunteers, researchers, business owners and non-execs in other organisations. In addition, we live intersectional lives that also impact on our lived-experience analysis, including in terms of age, gender, sexual orientation, class, physical disability and our refugee status.

We encourage you to read the paper, to digest it and to think about what you might wish to do as a result of having explored these issues.

## Introduction

We are the Lived Experience Advisory Board (LEAB) at the ESRC Centre for Society and Mental Health (CSMH), King’s College London. Although they may go by different names, there are many similar advisory groups taking place in research projects and institutions, and many of us have been involved in others. We felt the need to make a statement about how we feel and think about lived experience (LE) and the value it has in mental health research. We hope, however, that it touches on the wider experiences of people with lived experience working in similar contexts, both within academia and beyond. We also hope it will resonate with people with other types of lived experience, for example care leavers or people with experience of the asylum system, among others. We want to share what lived experience in research means to us; we want to articulate the value of lived experience and its potential to change, challenge and enrich research; we want to encourage organisations to consider what they need to do to listen to and learn from lived experience. We want to showcase the breadth and depth of lived experience whilst highlighting the skills and knowledge we bring to research. This paper covers a number of aspects of lived experience involvement including understanding lived experience terminology, experiential knowledge, individual and collective knowledge, power dynamics and organisational readiness. Our overarching argument is that lived experience involvement is crucial to creating high-quality, responsive mental health research that is seen as trustworthy and credible by those whom it is mean to benefit, but that this can only be achieved if universities and other organisations are committed, prepared and adequately resourced to involve people with lived experience in meaningful, ethical and equitable ways.

This paper will be a valuable resource for a number of audiences:

- people with lived experience who are involved in research, or are thinking of becoming involved,
- researchers and research centres working to involve people with lived experience in authentic and equitable ways.
- third sector lived experience organisations who are supporting people with lived experience and researchers to work together,
- the higher education sector more broadly, including funding bodies and policymakers.

We are passionate about making a difference, about having an impact. This paper is just the beginning. We would like to reach a point where everyone involved in research, from all backgrounds, understands the value of involving people with lived experience.

**Table 1:** **About us: the Lived Experience Advisory Board at the ESRC Centre for Society and Mental Health, King’s College London**

As a group, we have direct and/or indirect experiences of disabling barriers, neurodivergence, mental distress, mental illness, trauma, caring/supporting people in mental distress, and/or (ref)using mental health services including experiences of iatrogenic harm. We also hold intersectional identities in terms of our age, gender identity, class, ethnicity, and sexual orientation and see this as a key strength of our group. We seek to challenge a) traditional hierarchical power structures/systems and b) notions of what it means to “hold or produce knowledge,” both within academia and beyond. We came together in a series of workshops to share our personal views and experiences on the value of lived experience in research with the aim of sharing our views with others.	

## What do we mean by lived experience?

### A note on language

In the context of mental health research, the term ‘lived experience’ is used to describe people who have experienced mental distress, trauma and abuse and/or (ref)using services and treatments. The term is not universally used, and those who have been involved in mental health research or services for a long time will know that other terms have been used in the past, for example: service users, survivors, experts by experience, consumers.

Many of us have our own preferred language or terminology and it is impossible to find a term that everyone likes. Some prefer to use ‘living experience’ to reflect the ongoing nature of mental distress. Some prefer to use ‘mad’ because it is a term that has been reclaimed within the context of mad activism. And some prefer to use ‘survivor’ and ‘survivor researcher’. Others prefer ‘experts by experience’ as it sounds more empowering. It is important to ask people what term/s they prefer.

Unfortunately, due to the generic nature of the term ‘lived experience’, it can and has been widely adopted and co-opted, misused and exploited. We have seen people incorporating lived experience in ways that are disingenuous and do not actively involve diverse voices in order to tick the ‘lived experience’ box. We have also witnessed people in positions of power in research reveal that they have lived experience and using that to tick the ‘lived experience’ box without involving others.

### Understanding lived experience

It is important to remember that everyone’s lived experiences are different. In Box 1, we shared the breadth of lived experience that we bring to the Lived Experience Advisory Board. When we talk about lived experience, we are talking about the experience of pain and distress, in a context of social inequality and oppression, and in the context of often damaging mental health services. Lived experience is not about diagnosis; some people do not identify with the diagnoses they have been given and others do; it is not something we have much control over.

Some people’s distress is so severe or public that they have no choice about being drawn into the mental health system; some people try to avoid services altogether. Some of us will be more or less affected by a combination of gender, race and class (amongst other factors), all of which might increase our experiences of discrimination and disadvantage, both within mental health services and in wider society. Sometimes, lived experience involvement can be conflated with equality, diversity and inclusion, and this can mean that we are expected to represent the entire spectrum of lived experience. In reality, we each have our own intersectional positionalities (our own unique experiences), and working towards equality, diversity and inclusion should be everyone’s responsibility (not just those of us with lived experience).

Lived experience of mental distress is also about the experience of every-day inequality. Many of us experience people seeing us or treating us as ‘other’ or believing our views to be of less value. The implication or assumption that we are inferior does not just cause us harm, it also means that our experiences and views are left out of academia. This is significant as without the inclusion of lived experience, research is incomplete.

## How do we move from our individual lived experience to influencing and impacting research?

We have some responsibility to move from our individual lived experience to being in a position where we can influence and contribute to the work of a research centre like the ESRC Centre for Society and Mental Health. This is a learning, iterative process where we aim to transform our experiences into knowledge by collating our experiences with the experiences of others who have experienced mental distress, and placing them in their broader social, cultural and political contexts. This process of moving from individual experience to making a meaningful contribution can be enabled through peer support (a community of practice), training and supervision. Meeting with each other, learning from each other’s experiences and skills can be empowering. Having the space to meet, to develop and form strong connections as a group is an important part of this process. This means that while payment is important, it is not sufficient by itself; we should also have access to support, safety, training and skills development opportunities [10]. 

We discussed at length the dilemma of being involved because of our lived experience and then having our skills, opinions or expertise diluted, devalued or dismissed as a result. The very reason why we have been included is also the grounds for why our input is subsequently ignored. Sometimes this is because people assume that we only have our lived experience to offer, when we have other skills to offer too - someone referred to this as ‘loony overshadowing’!

We talked about anger and its relationship with the passion for change and connected this back to our experiences of powerlessness and inequality. We feel that everyone needs to hear and acknowledge the anger that emerges from time to time, although it can be hard for those who are unused to it. The ability to listen to the essence of what is being said, to not take it personally, and to lean in to be able to find out more is a skill that can be learned. Some professionals prefer to involve people who are quiescent, who do not challenge them, but instead act as ‘rubber-stamps’ to the traditional decisions and processes. But, if we are to have meaningful impact, we have to be able to express that passion. One member of the group said:

*It’s almost like you have to take the hand of the people in power and say ‘let me take you to the edge of how bad things are*,* and don’t look away. You need to see this to understand how bad things are’. They won’t go there by themselves.*

We want the relationship between researchers and people with lived experience to be different: we want to be seen as having expert knowledge alongside other skills, rather than as passive recipients of care and services. The aim would be to change people’s relationship with us in ways that can be empowering for us and enlightening to them.

## **What value does lived experience bring to research?**

We firmly believe that, as people with lived experience, we bring value to research and to the ESRC Centre for Society and Mental Health. As a group, we want to see research that makes a difference – to help all of us to create new knowledge that will have positive outcomes for people’s mental health and wellbeing. In our discussions, we quoted the slogan ‘Nothing about us without us’, a challenge that originated from the Disability Movement. It conveys the idea that no policy should be decided by any representative without the full and direct participation of members of the group(s) affected by that policy. Making a difference to research has the potential to make a difference for people with lived experience somewhere down the line.

We discussed the multi-faceted nature of the value that we bring and have highlighted four key areas where we think our contributions are particularly strong.

### Accountability

We ‘stay with the trouble’, which means that [5]:

We can hold a mirror up to organisations to highlight the difference between what they say they do and what they actually do.

We are able to sit with feelings that many cannot sit with – we can sit with other’s distress without wanting to solve it or run from it.

We have the ability to hold complexity and not strive for simplistic explanations over nuance.

### Perspective and Insight

We can bring the results of having reflected extensively on our experiences – the intersectional oppressions, social issues, and mental health services.

Our hybrid positionality as both outsiders and insiders within research culture means that we are in a position to see patterns, gaps, and power dynamics that impact on individuals, projects and organisations.

By continually re-grounding research in experiential knowledge, we can ensure that research holds relevance and meaning for the people it is most likely to affect.

### Inclusion and Impact

We have experience and expertise in making things accessible, because of the communities we interact with.

We can connect with wider communities of people with lived experience beyond research programmes and centres.

We can have a clearer focus on impact because of the motivation to improve things for us and our peers.

### Inspiration

We bring energy and enthusiasm to our work.

We can support others with lived experience to express themselves.

In the UK, creating positive research cultures is becoming more important. What we mean when we talk about research cultures is not always clear. Sometimes, people mean things like transparent leadership, valuing diversity, seeking positive collaborations, and supporting individuals to feel safe and secure, but it can be broader than this too [7]. Many of us are thinking about how we can improve the research cultures we are part of. We note that the upcoming REF 2029 will include a heightened focus on research culture as something to be evaluated in universities [8]. This creates an opportunity for the inclusion of lived experience to be emphasised as a crucial part of building a positive research culture. We believe that for lived experience involvement to be meaningful it has to be present not only in the research itself, but at all levels of decision making within the research cycle including what goes on before and after, including shaping funding priorities on the front end and influencing policy and practice on the back end. There are several ways in which involving people with lived experience has the potential to lead to a more positive research culture. For instance, as a group, we tend to work in relational ways, prioritising the process and the connections with each other - we encourage a culture of listening and being willing to ‘unlearn’.

This way of working has enabled us to achieve a significant amount of meaningful impact with the ESRC Centre for Society and Mental Health and beyond. We are a core part of the governance structure of the Centre and have played an integral role in developing and operationalising the Centre’s Code of Conduct; we have engaged in a range of knowledge exchange activities sharing our ideas about best practice for lived experience involvement and inclusive working practices more broadly; and we have submitted evidence to several parliamentary enquiries where our evidence has been cited multiple times in the resulting government reports. These are just a few examples of how we have proactively shaped research culture and initiated and delivered our own impact projects, which we hope demonstrates that lived experience involvement can and should go beyond mere consultation and feedback [2, 3, 6]. 

## The nature of knowledge

One of the central goals of our presence within research is to challenge the nature of knowledge. People with lived experience have traditionally been excluded from creating the knowledge that is used to make decisions about our lives, treatments and services. We believe in the value of *experiential knowledge* – that is, the knowledge gained through living and lived experience. Borkman first conceived of experiential knowledge as ‘the truth learned from personal experience with a phenomenon rather than truth acquired by discursive reasoning, observation, or reflection on information provided by others.’ [1] Experiential knowledge goes beyond the individual experience through a process of analysing and reflecting on those experiences with other people and/or through reading and connecting with online communities. In this way, we learn to situate our individual experiences both socially and politically in relation to the experiences of others.

*‘As we discover how particular experiences are mediated through social relations*,* we can connect the ‘immediate’ experience we started with to the larger social organization*.’ [4].

Experiential knowledge is vital to the understanding of mental distress, trauma and the broad circumstances surrounding people with lived experience in society today. According to Taggart (2018), it is ‘*simply different and necessary to reach the corners of madness that science cannot’.* Without experiential knowledge, research has the potential to miss significant issues or features that might impact our understandings, the implementation of services or use of interventions. To give an example from the past, research that resulted in the widespread use of anti-psychotic medication failed to explore the unwanted effects of the drugs, which in turn resulted in many people stopping or not taking the drugs. Arguably, this in turn led to research into ‘compliance therapy’ in order to find ways of encouraging people to take the medication.

## The importance of power

We talked a lot about power, and the role it plays in our experiences and in our lives. When we talk about people with lived experience, we believe we have a shared understanding of what it means to be powerless, both within and beyond mental health services. Power operates in research contexts too, due to the academic hierarchy. People in positions of power have some responsibility to be aware of that power and to make efforts to be both transparent about the power they hold, and to share it along with the resources that underpin it.

It is also critical to be aware of who researches who: who has the power to direct the research and who is in the position of being researched. This is because, “In academic research there are powerful norms surrounding who gets to study whom” which lived experience involvement challenges; this can lead to powerful resistance [9]. 

As people with lived experience, we also have power in some situations. If a researcher or research project needs us to be involved in their application in order to apply for funding, we have some power over entering into that relationship. This has implications especially for marginalised communities who have strong reasons for not getting involved in research.

We agreed that it is essential to discuss power, and for all parties to be transparent with each other about where decisions are made and what decisions can be influenced – where the power lies and how that power is going to be used.

Power differentials can exist within lived experience spaces as well, not just between researchers and people with lived experience, and this can impact whose voices are heard, what opportunities are available to whom and ultimately whose perspectives are more likely to influence research directions and outcomes. As we’ve noted earlier, in our LEAB we hold intersectional identities in terms of our age, gender identity, class, race, ethnicity, and sexual orientation and see this as a key strength of our group. There are considerable differences within our group in professional backgrounds, academic familiarity, communication and working styles, trauma history, income security, to name but a few areas where our experiences diverge. In spite of these differences, we have been able to build a culture of trust and mutual respect, where we work together to achieve shared goals and create space for everyone to contribute in ways that work for them. We’ve achieved this by recruiting people who aren’t box-tickers, who perhaps haven’t felt at home in other spaces and who are action-oriented and committed to social justice. Together, we’ve created a space that is built on psychological safety, open dialogue, constructive healthy debate that leaves space for disagreement, vulnerability and admitting mistakes, bias for action and shared decision making and accountability. This is not always the case and it’s important for researchers and organisations to be conscious that people with lived experience are not a monolith and that inequalities can be replicated within lived experience spaces if not attended to.

### What do research programmes and centres need to do to be able to hear us and accept change to their work?

We agreed that people in projects and/or research centres need to consider why they are involving people with lived experience before doing so. Ideally, everyone should be made aware of the reasons for involving people with lived experience to reduce ambivalence. Everyone needs to be open to change and to being challenged.

**Table 2:** **Disruption**

One member of our group gave an example of a local council bringing in a ‘disruptor’ with the specific aim of disrupting the status quo. We feel that an appetite for disruption should be encouraged.	

It may be that some people, and some organisations, need preparation before bringing in people with lived experience. If people make the space for change to happen then the added value that people with lived experience bring can flourish. An openness to change is about allowing the space for disagreement. Involving people with lived experience should never be about ticking boxes or rubber stamping the work of academic researchers.

*We bring a combination of lived experience and critical friend. To get the benefit of the second*,* people need to accept the first.*

The role of being a critical friend is not an easy one and can bring emotional challenges for people on all sides. We talked about the need for support and transparency, and about the need to ensure that research projects never involve only one person with lived experience.

Researchers need to be clear that involvement in research, such as when individuals with lived experience are part of the steering group, is different from being a research contributor such as an interviewee or member of a focus group. We agreed that researchers should not question people with lived experience about their lived experience unless this is part of an interview for instance and this has been agreed. This can be difficult to negotiate: people may not necessarily feel safe to disclose their experiences or they might feel obliged to disclose at the time and feel emotionally exposed afterwards. People with lived experience should be invited to participate in research project management, to contribute their expertise and opinion, not to ‘tell their stories’.

We also discussed the potential for harm in sharing our lived experience in places where it is not heard or appreciated. It is important for organisations who genuinely want to hear from people with lived experience to build in some routes for support so that people can feel safe to share their experiences and to work together. Box 3 contains some features of organisations that are ready for lived experience involvement.

**Table 3:** **Is your organisation ready for lived experience involvement?**

These points are adapted from an activity posed by Charity Village: ***Is your organisation ready for a mentorship program***?	
•Senior leadership of the organisation is supportive.	
•Programme is led by a senior staff member.	
•The organisation has a culture of learning (and un-learning)?	
•Goals for the programme are clearly articulated.	
• There are resources (financial and staff time) that can bededicated to the programme.	
These points are articulated in full here:https://charityvillage.com/is_your_organization_ready_for_a_mentorship_program/Accepted manuscript	

**Some possible reflection questions for organisations and/or researchers are:**

1. What have you done to prepare yourself and your organisation for involving people with lived experience?
2. Are you clear what lived experience offers this project or research centre? If not, is there a risk of tokenism?
3. Are you including people who you know might disagree with you, and are you open to hearing critical things about your research?
4. Do you know how to respond to criticism, validating in a supportive way – or explaining why, if you cannot take their views on board?
5. Are you seeing people beyond their lived experience, recognising that they might have other skills?
6. Have you made space for people with lived experience to meet and support each other?
7. Have you secured resources to support lived experience involvement?

These prompts for reflection are just the beginning of the process, and we are aware that there are practical steps organisations will then need to take. To that end, we are developing an organisational readiness toolkit that will support researchers and organisations to develop the necessary skills and processes to enable meaningful lived experience involvement.

## Concluding remarks

We hope that you have enjoyed reading this paper. It is one possible first step towards taking action within your own organisation. There is much to learn regardless of whether you already involve people with lived experience in your work or not. We suggest that you use the paper as a discussion point in key places within your agency to explore what more you can do to create meaningful involvement that can liberate the potential of lived experience in social research organisations and beyond. The authors invite readers to continue this conversation with us by making contact via the corresponding author.

## Data Availability

No datasets were generated or analysed during the current study.
